# Aluminum-Crosslinked Nanocellulose Scaffolds for Fluoride Removal

**DOI:** 10.3390/nano14121032

**Published:** 2024-06-14

**Authors:** Ken I. Johnson, Sunil K. Sharma, Priyanka R. Sharma, Abdulrahman G. Alhamzani, Benjamin S. Hsiao

**Affiliations:** 1 Department of Chemistry, Stony Brook University, Stony Brook, NY 11790, USApriyanka.sharma@wmich.edu (P.R.S.); 2 Department of Chemical and Paper Engineering, Western Michigan University, Kalamazoo, MI 49008, USA; 3 Chemistry Department, College of Science, Imam Mohammad Ibn Saud Islamic University (IMSIU), Riyadh 11623, Saudi Arabia; agalhamzani@imamu.edu.sa

**Keywords:** nanocellulose, aluminum, ionic crosslinking, cationic adsorbent, fluoride removal

## Abstract

Anionic carboxylated cellulose nanofibers (CNF) are effective media to remove cationic contaminants from water. In this study, sustainable cationic CNF-based adsorbents capable of removing anionic contaminants were demonstrated using a simple approach. Specifically, the zero-waste nitro-oxidization process was used to produce carboxylated CNF (NOCNF), which was subsequently converted into a cationic scaffold by crosslinking with aluminum ions. The system, termed Al-CNF, is found to be effective for the removal of fluoride ions from water. Using the Langmuir isotherm model, the fluoride adsorption study indicates that Al-CNF has a maximum adsorption capacity of 43.3 mg/g, which is significantly higher than that of alumina-based adsorbents such as activated alumina (16.3 mg/g). The selectivity of fluoride adsorption in the presence of other anionic species (nitrate or sulfate) by Al-CNF at different pH values was also evaluated. The results indicate that Al-CNF can maintain a relatively high selectivity towards the adsorption of fluoride. Finally, the sequential applicability of using spent Al-CNF after the fluoride adsorption to further remove cationic contaminant such as Basic Red 2 dye was demonstrated. The low cost and relatively high adsorption capacity of Al-CNF make it suitable for practical applications in fluoride removal from water.

## 1. Introduction

The world’s supply of fresh water is under immense pressure as the global population rapidly expands. Today, approximately 2 billion people live without access to clean potable water [1]. Among varying water pollution issues, the problem of fluoride contamination in drinking water has become a notable challenge in some regions. While low doses of fluoride (around 0.7 ppm) can improve dental health by reducing the likelihood of cavities and tooth decay, high concentrations of fluoride in drinking water can lead to negative health effects such as dental and skeletal fluorosis, arthritis, bone damage, osteoporosis, and chronic issues [2,3]. The sources of fluoride pollution vary widely in different regions but are typically attributed to natural pollution and/or anthropogenic causes. Natural pollution is usually caused by fluoride salts that are held inside rocks and sediment, which can be released into the environment over time through weathering and other geological processes. In this case, regions dependent on ground water for drinking are particularly susceptible to the harmful effects of high fluoride concentrations. In contrast, fluoride pollution due to anthropogenic causes can range from the inappropriate use of agricultural chemicals to the mismanagement of industrial waste [4].

Recently, nanocelluloses, such as cellulose nanofibers (CNF) and cellulose nanocrystals (CNC), have been recognized as effective and sustainable nature-based water remediation materials, and they can be prepared from any lignocellulose biomass feedstock [5,6]. These materials not only retain environmentally friendly cellulosic properties, but also possess characteristics such as large surface area, abundant functionalities and scaffolding stability, thus offering new opportunities for a broad spectrum of water purification applications. The typical production of nanocellulose consists of combined chemical (e.g., from popular TEMPO-mediated oxidation [7] to the recent nitro-oxidation process (NOP) [8]) and mechanical (e.g., high pressure homogenization and ultrasonic) [6] treatments to fibrillate microscale fibers into nanoscale fibers. The conventional chemical treatments involve the conversion of primary hydroxyl groups in the anhydroglucose units of the cellulose chain into negatively charged groups such as carboxylate groups (by TEMPO and NOP treatments) that are capable of adsorbing cationic contaminants such as toxic metal and ammonium ions. However, the anionic nanocellulose scaffold is not effective at removing anionic contaminants, such as fluoride and nitrate ions.

In this study, we used a simple approach to convert anionic carboxylate nanocellulose into a cationic scaffold by crosslinking the nanocellulose with aluminum ions. The chosen nanocellulose was based on CNF produced from raw jute fibers using the zero-waste NOP approach [8]. The NOP approach can be understood by a simple analogy borrowed from the kitchen. To make vegetable soup, one starts with vegetables and water. When cooked, the nutrients are fused with the water and the vegetables are turned into insoluble pulp. In NOP, the essential ingredient, nitric acid, is the same basic chemical used to produce synthetic nitrogen fertilizers for farming. NOP works because nitric acid is one of the most widely used digestion reagents for the decomposition of organic matter, as well as a primary oxidant for the oxidation of cellulose. In the NOP treatment, the lignocellulosic component is simultaneously pulped (delignified) and oxidized, resulting in a carboxylated cellulose scaffold that can be electrostatically swollen and mechanically defibrillated in the nanoscale with low energy. As the processed effluent from NOP contains nutrients released from the feedstock, it can be neutralized into effective fertilizers to augment synthetic fertilizers. Consequently, the NOP approach represents a zero-waste process, where the production cost of CNF by NOP can be a fraction of that by conventional methods.

In this study, raw jute was chosen as a model non-woody biomass feedstock to prepare NOCNF. It possesses a looser structure and relatively lower lignin content, similar to many agricultural residues and natural fibers, which need less energy (and chemical treatments) to defibrillate. It has recently been pointed out that non-woody biomasses, although abundant, are relatively underutilized around the globe when compared to woods [6]. To convert carboxylated CNF into a cationic scaffold, we used aluminum ions as an ionic crosslinking agent. Aluminum is the most abundant metallic element in the Earth’s crust, and its +3 charge makes the ionic species an effective crosslinking agent to bind the cellulose nanofiber scaffold (the resulting composite scaffold is termed Al-CNF). The presence of aluminum species renders the scaffold a positively charged medium which can adsorb negatively charged contaminants such as fluoride from water.

We note that the removal of fluoride contaminants can be carried out through various techniques, including ion-exchange, adsorption, coagulation–precipitation/electro coagulation, and filtration techniques such as reverse osmosis [9,10,11]. The advantage of adsorption over other techniques includes its high removal efficiency and potentially low cost. One disadvantage over other techniques is a sensitivity towards pH and other competing salts which may be in solution [12,13,14]. Currently, the common adsorption materials include aluminum oxide and activated carbon, which generally offer low-cost and high adsorption efficiency features to remove fluoride. We argue that the approach of using Al-CNF as an adsorptive medium for fluoride removal may offer another cost-effective pathway that can complement the existing adsorbents, especially when CNFs are prepared using the zero-waste NOP approach. Furthermore, the use of Al-CNF can mitigate the risk of secondary contamination from the use of typical aluminum-based adsorbents (such as activated alumina nanoparticles) at neutral pH levels.

## 2. Materials and Methods

### 2.1. Materials

Untreated jute fibers were provided by Toptrans Bangladesh Ltd. (Dhaka, Bangladesh), Nitric acid (65 wt%), sodium nitrite (97%), aluminum sulfate hydrate (≥97 wt%), hydrochloric acid (1.0 N), and sodium fluoride (≥99%) were purchased from Sigma Aldrich, St. Louis, MO, USA. Sodium hydroxide (99%) was purchased from Macron Fine Chemicals, Thermo Fisher Scientific, Waltham, MA, USA. Sodium bicarbonate (99%) was purchased from Fisher Scientific, Waltham, MA, USA. All chemicals were used without further purification.

### 2.2. Preparation of Nitro-Oxidized Cellulose Nanofibers (NOCNF)

NOCNF were prepared using the following procedures. In brief, 1 g of untreated ground jute was placed in a 1 L round-bottom flask, to which 14 mL of nitric acid was subsequently added. When the samples became completely soaked in the acid, 0.96 g of sodium nitrite was added to the reacting mixture under continuous stirring. Upon the addition of sodium nitrite, red/brown fumes evolved inside the flask, which was sealed with stoppers. The reaction was performed at 50 °C for 12 h, and then subsequently quenched by adding 250 mL of deionized water to the flask. The resulting mixture was transferred to a beaker for decantation. After the solids settled, the supernatant liquid was removed and neutralized for fertilization study, which will be described elsewhere. The slurry was further washed with deionized water and the decantation step was repeated until the pH level of the upper supernatant was above 2.5. The fiber slurry was then transferred to a dialysis bag (Spectral/Por, Thermo Fisher Scientific, Waltham, MA, USA, MWCO: 6–8 kD) and equilibrated for 4–5 days until the conductivity of the wash water reached below 5 µS/cm. As the pH value of the resulting cellulose nanofibers was between 2.8 and 3.0 due to the presence of carboxylic acid groups, the agglomeration of fibers was seen during the dialysis. To convert the carboxylic acid groups into carboxylate groups, the resulting fibrous suspension was treated with 4 wt% sodium bicarbonate solution, until the pH level reached 7.5. The treatment produced carboxylate groups on cellulose chains and negative charges on the cellulose surface. To create nanofibers, the slurry was passed through a homogenizer at 250 bar for 1 pass to fibrillate the microfibers into NOCNF.

### 2.3. Preparation of Al-CNF Composite Suspension

The preparation of aluminum-crosslinked NOCNF suspensions was carried out as follows: 100 g of 0.294 wt% suspension of NOCNF was added to a 250 mL beaker under vigorous stirring using a one-inch Teflon coated stir bar. In this preparation, one mole of aluminum ion was added to one mole of the glucose unit in nanocellulose to ensure an excess amount of aluminum ions for crosslinking. In this scenario, with the 1.00 molar solution of aluminum ions from aluminum sulfate hydrate, 3630 µL was added dropwise to the NOCNF suspension. It was found that upon adding approximately half of the aluminum solution, a rigid gel formed immediately. Stirring was continued to break down the gel to allow for the continuing addition of aluminum solution. Upon the complete aluminum solution addition, the suspension was continuously stirred for an additional 3 h. Subsequently, the final suspension was centrifuged, then decanted, and the supernatant was removed. Deionized water was then added to the sample to resuspend the final product. This procedure was repeated until the supernatant of the suspension maintained a conductivity below 10 µS/cm. The final suspension sample was kept at 2–4 °C until further study.

### 2.4. Characterization of NOCNF and Al-CNF

#### 2.4.1. Conductometric Titration

In this test, 40 g of NOCNF suspension was diluted to 0.2 wt% using 160 g of water under stirring at a rate of 300 rotations per minute (rpm). The dilution process allowed the suspension to be stirred easily while avoiding gelation during the addition of salt solutions. The pH level of the suspension was then adjusted to 2.5 using dilute hydrochloric acid, and subsequently titrated with 0.05 M of sodium hydroxide in 1 mL increments until the pH value of 10 was reached (a more dilute sodium hydroxide solution was used in the late stage of the dilution).

The conductivity of the suspension was measured during titration and plotted against the volume of the titrant. The resulting graph usually exhibited three linear regions. From volume 0 to *V*_1_, the increase in titrant should decrease the conductivity until a low plateau volume (*V*_1_) is reached. This is the result of neutralizing the excess amount of free acid in the suspension. The further addition of titrant does not change the conductivity of the suspension because the process neutralizes protons bound to the carboxylic acid groups on CNF. This is true to a certain volume (*V*_2_) of titrant used, above which additional titrant introduces free hydroxide and increases the conductivity. The carboxylic acid concentration can thus be calculated using Equation (1) [8].

(1) $$\frac{mmol\;carboxylate}{g\;NOCNF(s)}=\frac{\left(V_{2}-V_{1}\right)\times C_{t}}{m}$$

where *C_t_* represents the titrant concentration. The numerator is typically converted into the unit of mmols. The symbol *m* represents the mass of solid NOCNF, which can be calculated by first converting the weight percentage (wt%) of the suspension into a fraction (i.e., the mass of solid NOCNF over the mass of suspension). The solid mass is then quantified by multiplying by the mass of original suspension used before dilution. The final carboxylic acid concentration represents the carboxylic acid content in mmols of carboxylic acid per gram of solid NOCNF.

#### 2.4.2. Fourier-Transform Infrared Spectrometry

Fourier-transform infrared (FTIR) spectroscopy was recorded on a Nicolet iS10 FT-IR Spectrometer by Thermo Scientific using the attenuated total reflectance (ATR) mode. Sixteen scans were averaged at a resolution of 4. The instrument was equipped with a DTGS KBr detector (Thermo Fisher Scientific, Waltham, MA, USA), KBr beam splitter (Waltham, MA, USA), IR source, Smart iTR accessory, and diamond window. Samples were generally measured from 400 to 4000 cm^−1^.

#### 2.4.3. X-ray Photoelectron Spectroscopy

X-ray photoelectron spectroscopy (XPS) was performed on a custom-built instrument. The instrument contained an X-ray source from PHI Electronics (Chanhassen, MN, USA), a spectrometer from V.G. Scientific CLAM 100 (VG Scientific, Waltham, MA, USA), and a VGX900I controller (Waltham, MA, USA) data collection system. The chosen X-ray source was Al Kα_1,2_, and the measurement was made under an ultrahigh vacuum of $1.0\times 10^{-8}$ torr. The measurement was collected at a 90° take-off angle relative to the sample film. The sample charging was corrected using the C1s peak of adventitious carbon (a thin layer of carbonaceous material on the surface of the air-exposed sample) at 284.8 mV. The Na KLL Auger peak was typically observed at 497 eV from the COONa group.

#### 2.4.4. Zeta Potential

The average zeta potential of the suspension samples was measured using a ZetaProbe Analyzer (Colloidal Dynamics, Palm Valley, FL, USA). The value was determined based on the assumption that the CNF has a dielectric constant of 5.0 and a density of 1.5 g/mL. In the measurement, aqueous suspensions were stirred at 300 rpm and titrated with an auto-burette. The instrument was equipped with a niobium electrokinetic sonic amplitude (ESA) electrode and was calibrated using a KSiW suspension. Additionally, samples were treated with either hydrochloric acid or sodium hydroxide using an auto-burette to achieve the desired pH value before the analysis.

#### 2.4.5. UV–VIS Analysis

A Genesys 10S UV–Vis Spectrophotometer (Thermo Scientific, Waltham, MA, USA) was used to measure the transmittance (%) of the sample in a quartz cuvette from 190 to 1100 nm using a 1 nm increment. The instrument was equipped with a Shimadzu SPD-M20A UV/VIS (Shimadzu, Kyoto, Japan) photodiode array detector.

#### 2.4.6. Scanning Electron Microscopy

Scanning electron microscopy (SEM) was carried out on a ZEISS Crossbeam 340 instrument (ZEISS, Oberkochen, Germany) using an electron high tension (EHT) of 3 kV and an accelerating voltage of 30 kV. Samples were prepared by casting a 0.1 wt% suspension of NOCNF on a silicon wafer, and then coating by gold sputtering. The instrument was also equipped with energy-dispersive X-ray spectroscopy (EDS) capability for elemental mapping. The EDS analysis was conducted in conjunction with SEM imaging.

#### 2.4.7. Transmission Electron Microscopy

Transmission electron microscopy (TEM) was conducted on a JEOL JEM 1400 instrument (JEOL, Peabody, MA, USA) using an accelerating voltage of 120 kV. The samples were prepared on 300 mesh copper grids (Ted Pella Inc., Redding, CA, USA) by casting 10 µL of a 0.01 wt% sample onto the grid. After removing the excess fluid, the sample was stained with 10 µL of 2 wt% aqueous uranyl acetate solution. The excess solution was removed, and the grid was left to air dry.

#### 2.4.8. Atomic Force Microscopy

Atomic Force Microscopy (AFM) measurements were performed using a Bruker Dimension ICON instrument (Bruker, Billerica, MA, USA). This instrument was equipped with a Bruker OTESPA tip (the radius was 10 nm). In sample preparation, 10 µL of a 0.005 wt% suspension was deposited on the surface of a silica plate and air-dried, and the measurement was carried out in the tapping mode.

#### 2.4.9. Wide-Angle X-ray Diffraction

A MiniFlex instrument from Rigaku, Japan, was used to measure the wide-angle X-ray diffraction (WAXD) patterns. The samples were measured from 5 degrees to 85 degrees in steps of 0.02 degrees, at a speed of 5 degrees per minute. The scan axis was set to theta/2-theta in a continuous mode, where the intensity was measured in counts per second. In this measurement, the voltage and current were set to 40 kV and 15 mA, respectively, using Cu Kα radiation. Additionally, an incident side and receiving side Soller slit of 5.0 degrees along with an incident-beam divergence-limiting slit of 1.250 degrees were used in the measurement.

#### 2.4.10. ^13^C and ^27^Al Cross-Polarization Magic-Angle Spinning Nuclear Magnetic Resonance

^13^C and ^27^Al cross-polarization magic-angle spinning nuclear magnetic resonance (CP-MAS-NMR) of Al-CNF were carried out using a Bruker Utrashield 500 WB plus (500 MHz) instrument (Bruker, Billerica, MA, USA). The instrument was equipped with a 2.5 mm triple resonance MAS NMR probe, capable of spinning the sample up to 35 KHz. The chosen resonance frequency was 10,000 Hz, and the samples were spun at the magic angle at a speed of 10 KHz.

#### 2.4.11. Thermogravimetric Analysis

Thermogravimetric analysis (TGA) was taken on a TA Q50 instrument (TA Instrument, New Castle, DE, USA). In this measurement, the sample was heated from 25 °C to 800 °C at a rate of 10 °C per minute under a nitrogen atmosphere. The sample was loaded on a platinum pan, where its mass was monitored as a function of temperature. The derivate weight, shown as %/min, was also plotted as a function of temperature, and its profile is typically referred to as derivative thermogravimetry (DTG). The DTG profile usually exhibits a clearer change in the weight loss.

#### 2.4.12. The Fluoride Adsorption Study

The fluoride adsorption capacity of Al-CNF was determined using different concentrations of sodium fluoride solutions (5 ppm to 2500 ppm), where the concentration was determined by a fluoride ion-selective electrode (ISE, San Diego, CA, USA, model number ISEF121, HACH, USA). In this study, 3 mL of Al-CNF suspension of known weight percent (wt%) was mixed with 3 mL of sodium fluoride of known concentration. After equilibrating for an hour, the samples were centrifuged at 10,000 rcf for 5 min to separate the solid and liquid phases. The supernatant portion was analyzed using the ISE and compared against the control samples (i.e., 3 mL of water and 3 mL of sodium fluoride solution with known concentration). To study the pH effect on the fluoride adsorption capacity, the pH value of the sample was systematically adjusted before the incubation period.

The methodology to determine the fluoride removal efficiency is as follows. The initial concentration of fluoride is the concentration of fluoride solution to be treated with Al-CNF (the fluoride solution was mixed with the Al-CNF suspension in a 1:1 volume ratio). The equilibrium fluoride concentration thus equals half of the initial fluoride concentration. All samples are then tested against the control solution (i.e., the 1:1 mixture of the same fluoride solution and water). The efficiency is defined as the percentage removal of fluoride between the control and the sample. Difference in concentration is the difference in the fluoride concentration between the sample and its respective control. As a result, the amount of fluoride removed represents the conversion of fluoride concentration into solid fluoride. The quantity of Al-CNF is derived from the weight percent (wt%) and the volume of Al-CNF suspension used. *Q_e_* represents the ratio between the fluoride removed and the quantity of Al-CNF used (mg/g).

Furthermore, mixed solutions of fluoride, sulfate, and nitrate were prepared to evaluate the selectivity of Al-CNF. For this purpose, a solution containing equal moles of three salts, sodium fluoride, sodium sulfate, and sodium nitrate, was prepared and then mixed with Al-CNF (1:1 by volume) and then left to equilibrate overnight.

## 3. Results and Discussion

### 3.1. Structure and Functionality of NOCNF and Al-CNF

**Figure 1:**
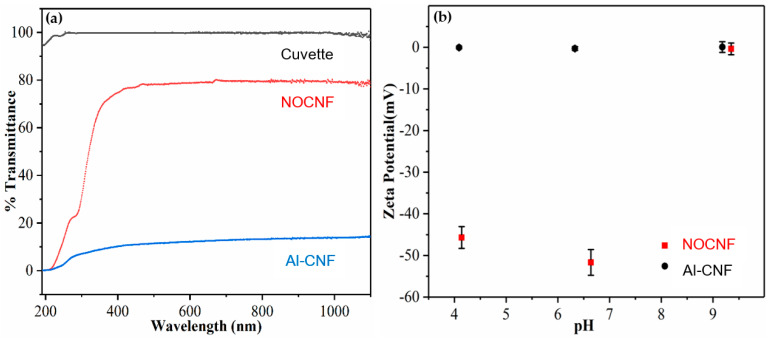
(**a**) The % transmittance curves of nanocellulose suspensions (0.5 wt%) containing NOCNF and Al-CNF as a function of the wavelength. (**b**) Zeta potential of corresponding NOCNF and Al-CNF suspensions as a function of pH.

It is interesting to observe that at the same concentration of 0.5 wt%, the Al-CNF suspension is much more viscous than the NOCNF suspension. With a slight increase in concentration, the Al-CNF system can readily form a stable gel with solid-like property. Figure 1a illustrates the transmittance curves of two suspensions based on NOCNF (0.5 wt%) and Al-CNF (0.5 wt%; to prepare this suspension, 250 mL of 0.5 wt% NOCNF suspension was mixed with 15.4 mL of 1.0 M aluminum solution using aluminum sulfate), respectively, as a function of the wavelength. Visually, the NOCNF suspension is relatively transparent, but the Al-CNF suspension is near-opaque. For example, at the wavelength of 800 nm, the % transmittance of the NOCNF suspension is 79%, but that of the Al-CNF suspension is only 13% (i.e., a six-fold reduction). This indicates that the scaffolds in Al-CNF are much more tightly bound, and some clusters may be formed. The presence of Al-CNF cluster can scatter the light and greatly decrease the transmittance. In contrast, the NOCNFs are relatively more homogeneously dispersed in the suspension, leading to a higher transmittance value. Figure 1b displays the zeta potential values of the NOCNF and Al-CNF suspensions as a function of the pH value, which was adjusted using dilute concentrations of hydrochloric acid or sodium hydroxide. This study confirms the anionic characteristics of NOCNF at the pH level below 7.0, where its zeta potential is in the range of −50 mV. At a high pH value (e.g., pH = 9.4), the zeta potential of the NOCNF is zero, indicating that it is not charged. In contrast, the zeta potential values of the Al-CNF suspension are seen to be around zero in the pH range tested (pH from 4.2 to 9.2, Figure 1b), indicating that the Al-CNF sample is in the neutral state. As the ionic bonding between the carboxylate group of NOCNF and the aluminum ion is very strong, it is not affected by the change in pH value.

**Figure 2:**
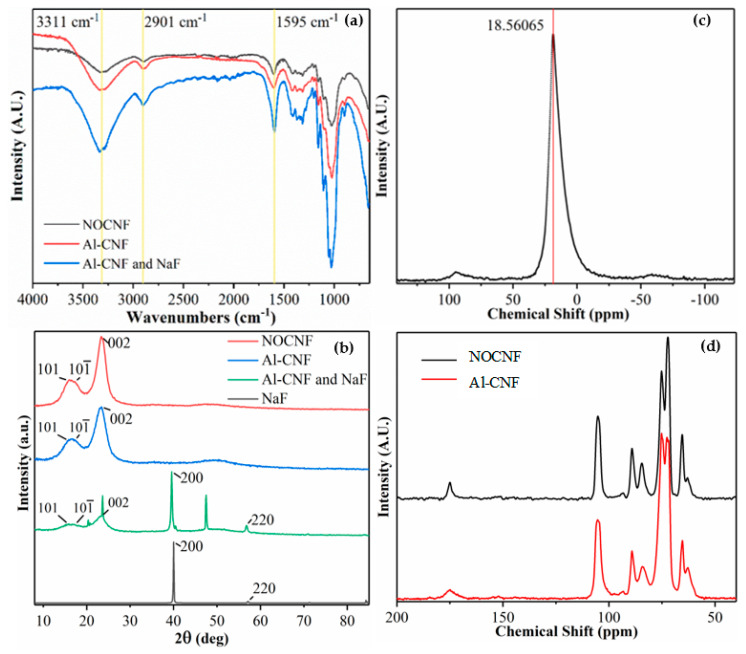
(**a**) The FTIR spectra of NOCNF, Al-CNF, and 1:1 Al-CNF mixed with 500 ppm of fluoride solution. (**b**) WAXD of NOCNF, Al-CNF, and 1:1 Al-CNF mixed with 500 ppm fluoride solution and sodium fluoride. The new peaks (24° and 48°) that appear in the Al-CNF/NaF sample indicate that crystallization occurs between aluminum and fluoride (e.g., the formation of aluminum fluoride hydrate crystals such as β-AlF_3_•3H_2_O). (**c**) The ^27^Al CPMAS-NMR spectra of Al-CNF (a major peak appears at 18.561 ppm). Additionally, (**d**) ^13^C CPMAS-NMR spectra of NOCNF and Al-CNF samples.

Figure 2a shows the FTIR spectra of dried NOCNF, Al-CNF, and the Al-CNF–sodium fluoride (NaF) mixture (from the 1:1 mixture of Al-CNF suspension and 500 ppm of sodium fluoride solution). All three samples exhibit the signature cellulose vibrational peaks, such as the carbonyl bond stretching at 1595 cm^−1^, C-OH stretching at 3311 cm^−1^, C-H stretching at 2901 cm^−1^ (all three characteristic peaks were indicated by yellow lines in Figure 2a), and C-O stretching at 1035 cm^−1^ [15]. It is interesting to note that the 1595 cm^−1^ becomes slightly broader with the addition of aluminum, possibly due to the formation of aluminum–carboxylate ionic interaction, where this peak becomes relatively more intense due to the presence of sodium fluoride, which possesses a vibrational peak at approximately the same location.

Regarding the crystalline structure of varying samples, Figure 2b shows the WAXD profiles of dried NOCNF, Al-CNF, and Al-CNF/NaF mixture samples, and pure NaF crystals. Both NOCNF and Al-CNF samples exhibit the typical WAXD pattern of the cellulose I structure, in which the three major diffraction peaks at 2θ positions of 16°, 18°, and 24° can be indexed by the (101), $\left(10\bar{1}\right)$, and (002) planes, respectively [16]. This confirms that the cellulose I crystal structure is maintained after NOP treatment. In the Al-CNF/NaF mixture, several new peaks appear (2θ positions at 20°, 24°, 40°, 48°, and 57°). The peaks at 2θ positions of 40° and 57° can be indexed by the (200) and (220) planes of the NaF crystal, respectively. The formation of the NaF crystal is due to the presence of excess NaF solution in the Al-CNF scaffold after being dried. However, the 20°, 24°, and 48° peaks that appear are not from the NaF crystal structure. This indicates the formation of a new crystal structure, probably from the interactions between aluminum and fluoride ions, such as the formation of aluminum fluoride hydrate crystals (e.g., β-AlF_3_•3H_2_O).

Al-CNF was analyzed using ^27^Al CPMAS-NMR, and the result is shown in Figure 2c. In this figure, a distinct chemical shift in the aluminum species at 18.6 ppm can be observed, which is consistent with the known range for AlO_6_ [17]. This is expected, as the aluminum ion can coordinate with the oxygen atoms in molecules of water, hydroxide, alcohol, and carboxylic acid. Specifically, Al^3+^ can form up to six coordinate bonds with these oxygen-containing groups in an octahedral arrangement. The ^13^C CPMAS-NMR spectra for NOCNF and Al-CNF are shown in Figure 2d. Both spectra exhibit a similar structure: the peaks at 63–65 ppm are due to the C6 primary alcohol, peaks at 72–75 ppm are due to the C5, C3, and C2 carbons, peaks at 84–89 ppm represent the C4 carbon, and the peak at 105 ppm represents the C1 carbon. These features have been explained in our previous publication [8]. Perhaps the only feature difference between the two spectra is the peak at 175 ppm due to the C6 carbonyl carbon. The slight broadening of the 175 ppm in Al-CNF is due to the bonding between the aluminum and carboxylate groups.

**Figure 3:**
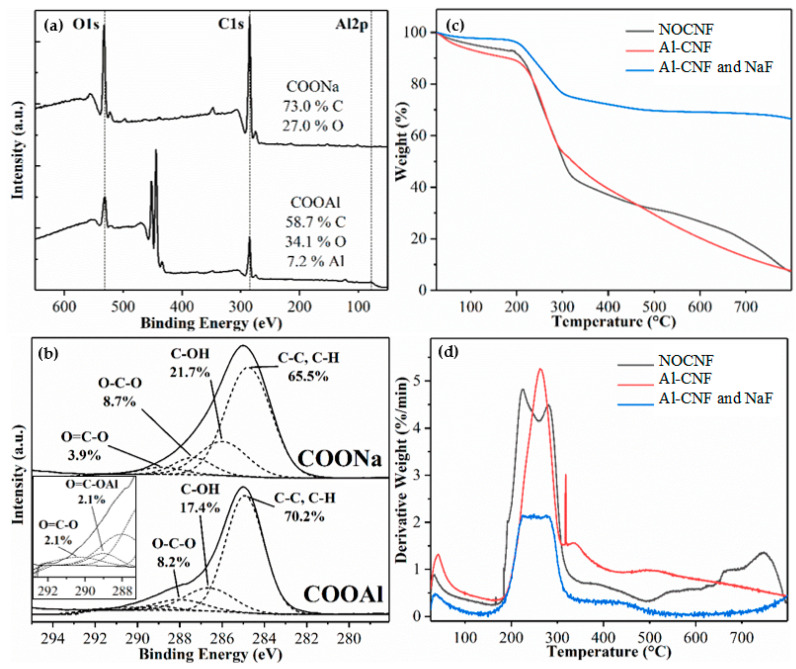
(**a**) XPS scans of NOCNF (indicating the COONa group) and Al-CNF (indicating the COOAl group). (**b**) The C1s peak in XPS associated with the COONa and COOAl groups, and the corresponding peak deconvolution showing relative concentrations of different bonds. (**c**) TGA profiles of NOCNF, Al-CNF, and Al-CNF/NaF mixture. (**d**) Corresponding DTG of NOCNF, Al-CNF, and Al-CNF/NaF mixture.

XPS scans of NOCNF and Al-CNF samples are shown in Figure 3a, which displays the elemental composition as well as the chemical and electronic states of the atoms within these materials. Using the sensitivity factor for C1s, O1s, and Al2p of 1.00, 2.93, and 0.753, respectively, the elemental composition of COONa was determined to be 73.0% carbon and 27.0% oxygen. Similarly, the elemental composition of COOAl was calculated to be 58.7% carbon, 34.1% oxygen, and 7.2% aluminum. The C1s peaks in Figure 3a associated with the COONa and COOAl groups were further deconvoluted, and the results are illustrated in Figure 3b. The C1s peak of COONa was deconvoluted into four peaks using a procedure including the application of a Shirley background, and fitting with four Gaussian functions constrained by peak position, peak area, and FWHM. The fitting results indicate the presence of C–C and C–H bonds (65.5%) corresponding to the cellulose backbone and hydrocarbon components, C–OH bonds (21.7%) corresponding to the carbon and hydroxyl bonds in cellulose, O–C–O bonds (8.7%) corresponding to the acetal groups in cellulose, and O=C–O bonds (3.9%) corresponding to the carboxylate groups. In contrast, the C1s peak of COOAl was deconvoluted into five peaks using the above procedures. The results indicate the presence of C–C and C–H bonds (70.2%) from the cellulose backbone and hydrocarbon component, C–OH bonds (17.4%) from the carbon and hydroxyl bonds in cellulose, O–C–O bonds (8.2%) corresponding to the acetal groups in cellulose, O=C–O bonds (2.1%) corresponding to the carboxylate groups, and a small peak due to the O=C–OAl bonds (2.1%) at around 288 nm (the inset in Figure 3b shows the deconvolution of the COOAl peak from 293 to 287 nm, where the results indicate the bonding between aluminum and carboxylate groups).

Figure 3c,d shows the TGA and DTG curves of NOCNF, Al-CNF, and Al-CNF/NaF mixture, respectively. All samples show that the onset temperatures of degradation begin at about 160 °C. The major peaks in the DTG curves for three samples occur in the range between 160 and 370 °C, representing the degradation of glucuronic acid and D-glucose in the cellulose chains. However, the TGA curves show that the associated weight losses for NOCNF and Al-CNF are similar and relatively large (about 60%), while that for Al-CNF/NaF is relatively small (about 30%). An additional peak at about 350 °C is seen in the DTG curve of Al-CNF, which may be a result of the CNF network crosslinked by aluminum with a slight increase in thermal stability. It is interesting to observe that the residual weight of Al-CNF/NaF is 68.9%. This may be largely due to the unabsorbed sodium fluoride crystals in the Al-CNF scaffold, which is consistent with the WAXD results in Figure 2b.

**Figure 4:**
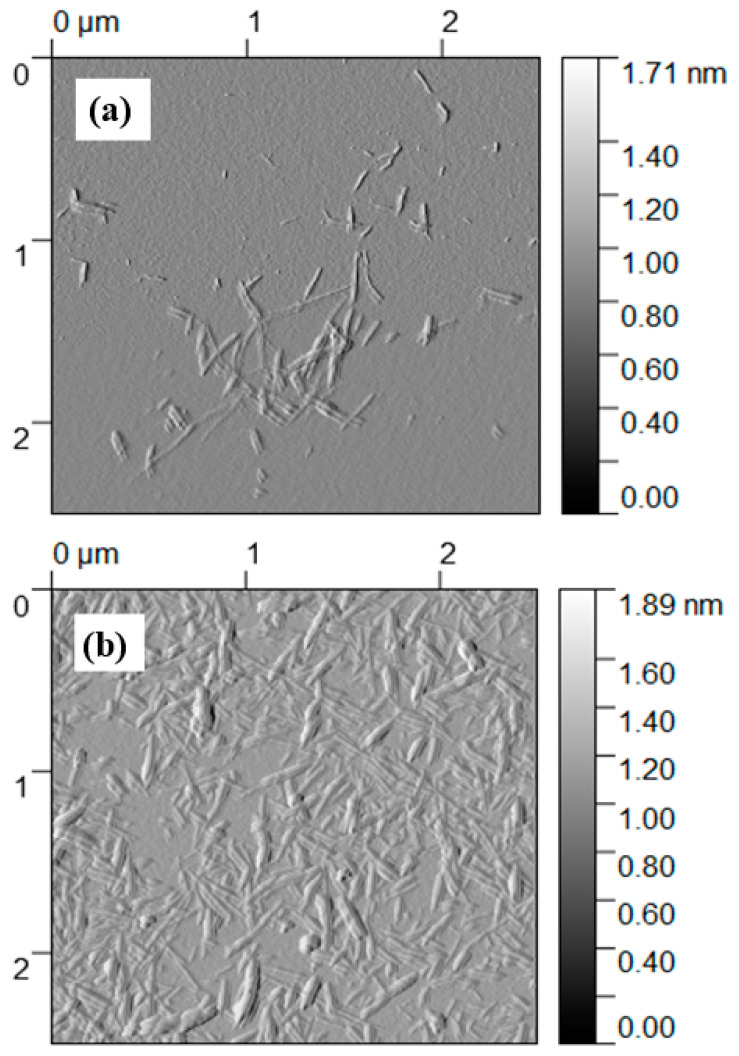
AFM images of (**a**) NOCNF and (**b**) Al-CNF samples cast and dried on the silica wafer substrate.

The morphology of the NOCNF, Al-CNF, and Al-CNF/NaF samples was investigated using several microscopic techniques. The AFM images of (i) NOCNF and (ii) Al-CNF samples cast on the silica wafer substrate are shown in Figure 4. It can be seen that the fiber length of NOCNF is relatively short, rendering its morphology more similar to that of CNC. A noticeable distinction is seen between the NOCNF and Al-CNF samples. In NOCNF, the fibers are relatively dispersed, and a higher degree of aggregation is observed in Al-CNF. Fiber aggregation behavior induced by the aluminum crosslinking interaction is expected in Al-CNF.

**Figure 5:**
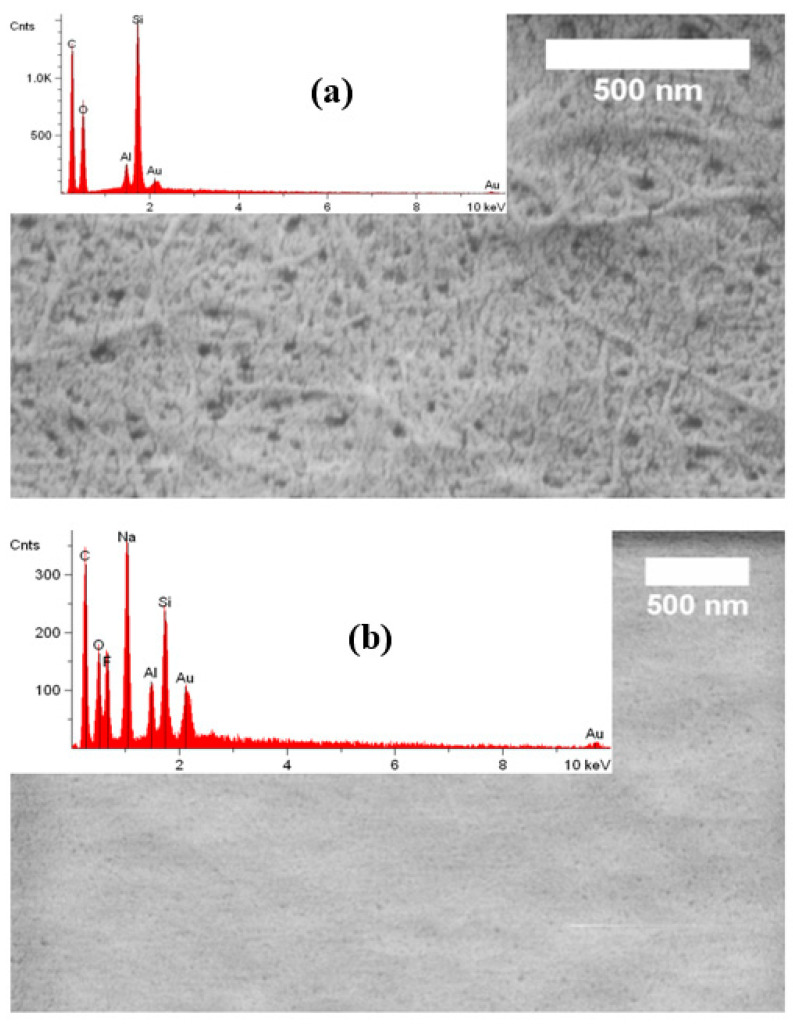
SEM images and corresponding EDS profiles of (**a**) Al-CNF and (**b**) Al-CNF/NaF samples.

**Figure 6:**
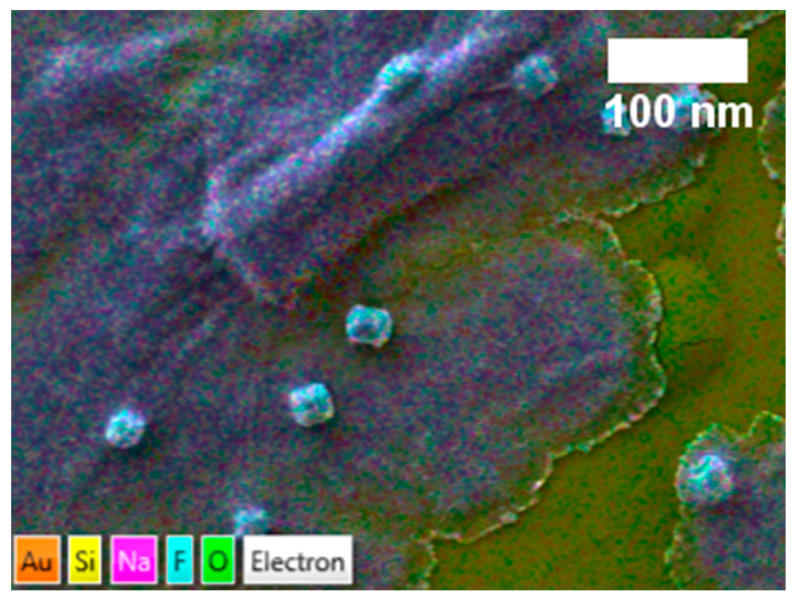
EDS color layered with the SEM image of the Al-CNF/NaF sample.

**Figure 7:**
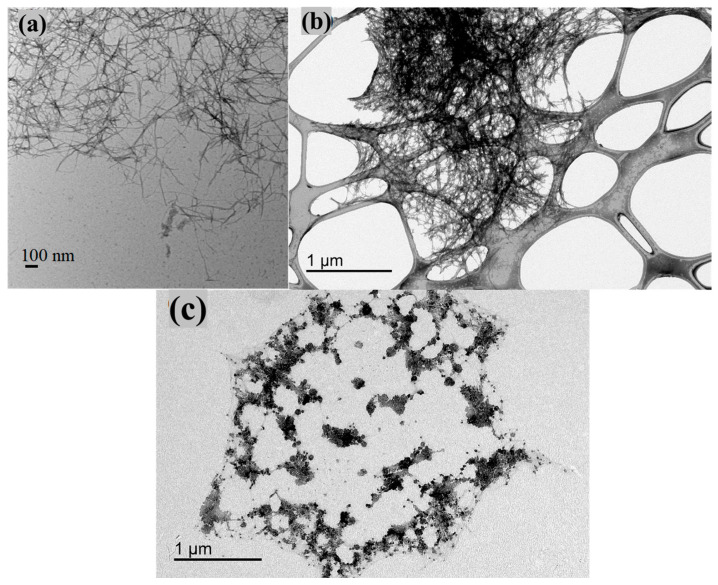
TEM images of (**a**) NOCNF, (**b**) Al-CNF, and (**c**) Al-CNF/NaF samples.

Figure 5 shows the SEM images and corresponding EDS profiles of Al-CNF and Al-CNF/NaF samples. In Figure 5a, the SEM image of Al-CNF exhibits a relatively porous structure due to the crosslinking effect of aluminum, causing a high degree of local CNF aggregation. The corresponding EDS profile confirms the presence of aluminum with no signs of sodium or sulfur, corroborating the completion of ion exchange by the adopted NOP approach. The presence of silicon comes from the substrate and gold from the sputter coating. In contrast, the SEM image in Figure 5b of Al-CNF/NaF exhibits a relatively smooth surface structure with low porosity. This is consistent with the adsorption of fluoride in the Al-CNF scaffold, and the formation of free NaF crystals. The corresponding EDS profile in Figure 5b confirms the presence of sodium and fluoride elements, where the relative low fluoride concentration (as compared to the sodium concentration) indicates that some fluoride was adsorbed and hidden in the Al-CNF scaffold. This hypothesis is verified by the EDS elemental colored SEM image of the Al-CNF/NaF sample, with (1) discrete NaF nanocrystals with a cubic structure (~20 nm) resulted from a cubic motif where both Na^+^ and F^−^ occupy octahedral coordination sites, and (2) sodium-rich Na and F nanostructures (probably not in the crystal form) which can be seen in Figure 6. The TEM images showing the fine morphological structures of NOCNF, Al-CNF, and Al-CNF/NaF samples are illustrated in Figure 7. The TEM images are consistent with the AFM images, which indicates the relative length of nanofibers, and the aggregation of nanofibers in Al-CNF. The TEM image of the Al-CNF/NaF mixture shows an even more aggregated structure, containing discrete black nanoparticles (NaF nanocrystals) and dark thin films covering the nanofiber texture. This thin-film structure appears to contain Al-CNF scaffolds which adsorb fluoride ions and are then further covered by sodium ions (as in Figure 6).

### 3.2. Fluoride Adsorption Study Using Al-CNF

**Figure 8:**
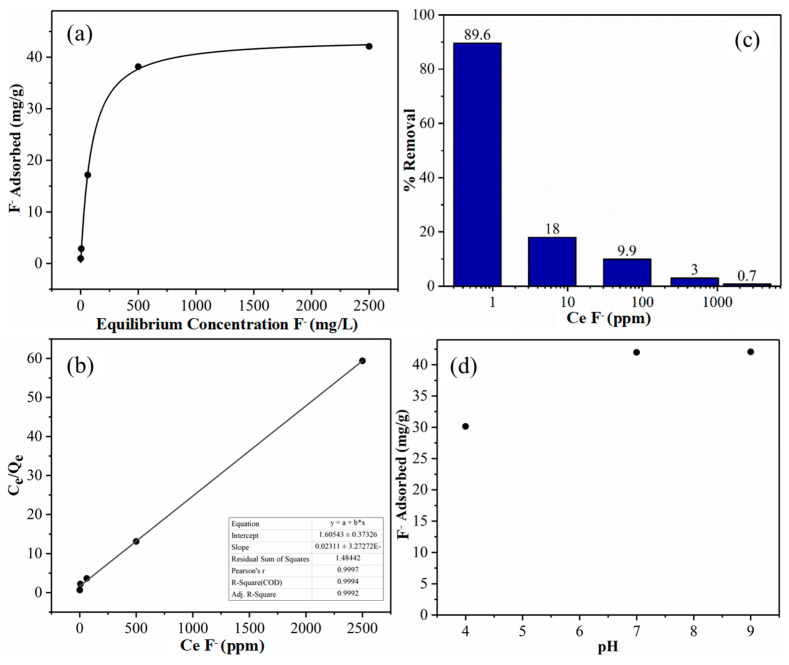
(**a**) Adsorption capacity of Al-CNF as a function of the fluoride concentration with the Langmuir isotherm fitting. The maximum experimental adsorption capacity is 42.1 mg/g measured at an equilibrium concentration of 2500 ppm fluoride solution. (**b**) The Langmuir isotherm fitting curve, where *C_e_*/*Q_e_* is plotted over C_e_ with an R^2^ value of 0.999. A maximum theoretical adsorption capacity (*Q_m_*) can be calculated to be 43.3 mg/g. (**c**) Percentage removal of fluoride using Al-CNF with varied equilibrium fluoride concentrations. (**d**) The adsorption capacity of Al-CNF at pH = 4, 7, and 9.

The fluoride adsorption study of Al-CNF was carried out using the sodium fluoride solutions of different concentrations. The experimental results are summarized in Table 1. Based on these results, Figure 8a shows the experimental adsorption capacity (*Q_e_*) in the mg/g of Al-CNF plotted against the equilibrium fluoride concentration (*C_e_*) in mg/L. In this figure, it can be seen that the fluoride adsorption capacity increases with the equilibrium fluoride concentration until the adsorption process reaches a plateau value (or asymptote). This plateau value (or the maximum experimental adsorption capacity) is 42.1 mg/g. Figure 8a indicates that with an increase in equilibrium fluoride concentration, the aluminum ions (cross-linkers) in the Al-CNF scaffold can further bond to fluoride ions, resulting in rapid uptake at the initial adsorption stage. As the active aluminum sites in Al-CNF become fully occupied, the scaffold becomes saturated and can no longer adsorb additional fluoride ions, resulting in the plateau region. We hypothesize that fluoride adsorption follows the monolayer adsorption process, which can be described by the Langmuir isotherm model with the following expression [18,19].

(2) $$\frac{C_{e}}{Q_{e}}=\frac{C_{e}}{Q_{m}}+\frac{1}{Q_{m}b}$$

where $Q_{e}$ represents the equilibrium adsorption capacity, $C_{e}$ is the equilibrium concentration of fluoride, $Q_{m}$ is a constant representing the maximum adsorption capacity, and *b* is a constant related to free adsorption energy. According to Equation (2), *Q_m_* can be determined from the inverse slope of the Langmuir plot (i.e., the plot of *C_e_*/*Q_e_* against *C_e_*), which is shown in Figure 8b. This figure exhibits a good linear relationship with an R^2^ value (0.999), which indicates the validity of the Langmuir isotherm model. We also used the Freundlich isotherm model, assuming the adsorption follows the multilayer deposition pathway to analyze the isotherm results, and the analysis results were not as good (the R^2^ value was around 0.98) as those from the Langmuir isotherm model. This indicates that the adsorption mechanism follows the monolayer deposition pathway, which is consistent with the prior hypothesis that the mechanism of fluoride removal is a chemisorptive ligand exchange reaction involving the formation of the internal complexation of fluoride with aluminum [20].

Based on the Langmuir fitting analysis, the maximum adsorption capacity (*Q_m_*) of Al-CNF is 43.6 mg/g. Compared to other published adsorbent materials used for fluoride removal, Al-CNF performs relatively well; the comparison is shown in Table 2. It can be seen that Al-CNF outperforms most naturally occurring inorganic minerals, such as alumina. Additionally, Al-CNF tends to do better than graphite, carbon nanotube, or chitosan-based materials. While some synthetic organic adsorbents (e.g., glutaraldehyde-crosslinked calcium alginate) can outperform Al-CNF, Al-CNF possesses the characteristic of better sustainability.

In Figure 8c, the percentage removal is seen to be inversely related to the equilibrium fluoride concentration. At low fluoride concentrations, Al-CNF offers plenty of aluminum binding sites where most of the fluoride can be removed from water (e.g., at an equilibrium fluoride concentration of 0.625 ppm, the percent removal is 89.6%, but the adsorption capacity of Al-CNF is only 0.96 mg/g). With the increasing fluoride concentration, the total amount of fluoride that is removed becomes greater, but the percentage removal is less because the Al-CNF scaffold becomes saturated.

Figure 8d depicts the adsorption capacity of Al-CNF at pH = 4, 7, and 9. It can be seen that the adsorption capacity remains constant at 43 mg/g for pH = 7 and 9, but decreases to 30 mg/g at pH = 4. Table 3 shows the concentration of aluminum in solution after the fluoride removal study at pH = 4, 7, and 9. It can be seen that at pH = 7, the smallest aluminum concentration of 2.7 ppb is detected, followed by 11.8 ppb at pH = 9 and 560 ppb at pH = 4. This study indicates that Al-CNF is not effective in acidic conditions. In other words, at pH = 4, a significant amount of aluminum can be dissolved, and the fluoride adsorption capacity of Al-CNF then decreases rapidly. This is because the carboxylate groups are more likely to bind with protons than aluminum ions at low pH values. This will be a limitation for the application of Al-CNF in acid conditions. However, the system of Al-CNF is quite effective for fluoride removal in neutral and basic conditions.

The zeta potential value of Al-CNF (Figure 1b) in the range of pH 4–9 is about zero, which indicates that Al-CNF is not charged in the suspension. The most plausible mechanism for the F^−^ sorption process is ion exchange, i.e., some of the aluminum–carboxylate bonds (on NOCNF) are replaced with alumina–fluoride bonds. Some researchers have reported that anions such as SO_3_^2−^, CO_3_^2−^, and NO_3_^−^ can be replaced with fluoride ions [28], which should also be the case for COO^−^. This hypothesis is certainly consistent with the formation of aluminum fluoride hydrate crystals (e.g., β-AlF_3_•3H_2_O).

### 3.3. Sequential Applicability and Comparative Studies of Al-CNF

**Figure 9:**
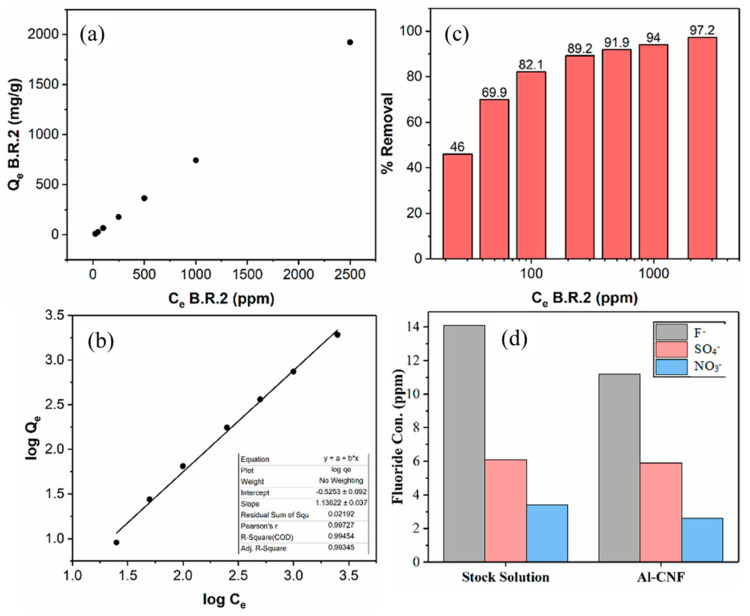
(**a**) The adsorption capacity of Basic Red 2 using the flocculated fluoride-adsorbed Al-CNF adsorbent. The maximum experimental adsorption capacity is 1923 mg/g using 0.126 wt% flocculated Al-CNF and 1:1 5000 ppm (initial) Basic Red 2. (**b**) The Freundlich isotherm fitting curve of Basic Red 2 removal using the fluctuated fluoride-adsorbed Al-CNF adsorbent. (**c**) Percentage removal of Basic Red 2 using flocculated fluoride-adsorbed Al-CNF. (**d**) The adsorption study using mixed ions of fluoride, sulfate, and nitrate. The stock solution is compared with the solution treated with Al-CNF.

It does not appear that Al-CNF can be regenerated and reused like other adsorbent materials. This is because, in the conventional regeneration process using acid, Al-CNF can release aluminum, which would make the system inefficient. To enhance the sustainability of Al-CNF, we have investigated the sequential applicability of fluoride-adsorbed Al-CNF for the possible removal of other cationic pollutants. To test this hypothesis, a cationic Basic Red 2 dye was chosen to test the removal capacity of flocculated fluoride-adsorbed Al-CNF (treated with a 500 ppm fluoride solution). Figure 9a shows the adsorption capacity of Basic Red 2 using fluoride-adsorbed Al-CNF. It is interesting to see that even at a 2500 ppm equilibrium concentration, the adsorption capacity of fluoride-adsorbed Al-CNF against the Basic Red 2 dye does not reach the plateau value, indicating that the removal efficiency can be even higher. Using 0.126 wt% of fluoride-adsorbed Al-CNF at a 1:1 ratio to a 5000 ppm initial concentration of Basic Red 2, the maximum experimental removal efficiency was found to be 1923 mg/g (i.e., *C_e_* at 2500 ppm in Figure 9a). We hypothesize that the isotherm adsorption data (Table 4) cannot be described by the Langmuir isotherm model, i.e., the adsorption mechanism does not follow a monolayer deposition process. In this case, we consider the use of the Freundlich isotherm model, assuming that the adsorption follows the multilayer deposition pathway. The expression for the Freundlich isotherm model can be described as follows [29,30].

(3) $$\log Q_{e}=logK_{F}+\frac{1}{n}logC_{e}$$

where *Q_e_* represents the adsorption capacity at equilibrium, *C_e_* represents the equilibrium concentration of the adsorbate, and *K_f_* and n are constants where *K_f_* is calculated as the y-intercept when plotting a linear curve of log*Q_e_* over log*C_e_*. The results of the Freundlich isotherm fitting curve for the Basic Red 2 removal using the fluctuated fluoride-adsorbed Al-CNF adsorbent are shown in Figure 9b. It can be seen that a good linear fitting of the experimental results using the Freundlich model (R^2^ is greater than 0.99) is found, again validating the selection of this analysis. In Figure 9c, the percentage removal is seen to increase with the equilibrium Basic Red 2 concentration. The results indicate that the adsorption of the Basic Red 2 molecules by the fluoride-adsorbed Al-CNF may not only be through electrostatic interaction, and that hydrophobic interaction and hydrogen bonding between the CNF scaffold and the dye molecules can also play a role. These effects will be explored in detail later. It is imperative to note that the concentration of aluminum after the dye adsorption has not been determined. This is important, considering the possibility of aluminum and fluoride losses. Nevertheless, the sequential applicability of the fluoride-adsorbed Al-CNF adsorbent for cationic contaminant removal can improve the sustainability of this absorbent system.

Finally, the selectivity of Al-CNF for anionic contaminant removal in the mixed solutions of fluoride, sulfate, and nitrate was investigated. The selection of nitrate and sulfate was made because both are common and enforceable regulated anionic contaminants, and nitrate is on the National Primary Drinking Water Regulations (NPDWR) standard list and sulfate is on the secondary regulation list. In this study, a solution containing three salts, namely sodium fluoride, sodium sulfate, and sodium nitrate, was mixed with Al-CNF (1:1 by volume) and then left to equilibrate overnight. The results are illustrated in Figure 9d, which shows that the fluoride concentration decreased from 14.1 ppm to 11.2 ppm, the sulfate concentration decreases from 6.1 ppm to 5.9 ppm, and the nitrate concentration decreased from 3.4 ppm to 2.6 ppm. The decrease in fluoride is very comparable to the results in Figure 8, indicating that the presence of other anions, such as sulfate and nitrate, does not significantly reduce the removal of fluoride. Such behavior has been reported in other aluminum-based adsorbents such as aluminum sulfate, alumina, and kaolinite [31,32]. This behavior can be explained because of how the different anions can bind with aluminum. It is known that fluoride binds with aluminum through an inner-sphere complex, while sulfate binds through both an inner- and outer-sphere complex, and nitrate binds through an outer-sphere complex [33]. Furthermore, non-ionic hydroxyls on the CNF surface have also been shown to form complexes with aluminum, such as polyphenols [34].

## 4. Conclusions

A new class of adsorbent, Al-CNF, prepared by the ionic crosslinking of an anionic nanocellulose scaffold produced from the zero-waste nitro-oxidization treatment of lignocellulose biomass, was demonstrated. The use of aluminum crosslinkers converts the anionic scaffold into a cationic adsorbent, which is effective for fluoride removal. The structure, functionality, and fluoride removal capability of Al-CNF were characterized by varying microscopic, spectroscopic, X-ray diffraction, and thermal analysis techniques. Using Langmuir isotherm modeling, the fluoride adsorption results from Al-CNF yielded the maximum adsorption capacity (*Q_m_*) of 43.6 mg/g, which is comparable or exceeds the fluoride removal performance of existing alumina adsorbents. However, Al-CNF exhibited some limitations in usage under the acidic conditions. The selectivity of fluoride removal in the presence of other anionic contaminants (e.g., sulfate and nitrate ions) remained high in neutral conditions. Finally, the sequential applicability of using spent Al-CNF after fluoride adsorption (i.e., fluoride-adsorbed Al-CNF) to further remove cationic molecules such as Basic Red 2 dye was demonstrated. The practice can further enhance the sustainability aspect of this already promising adsorbent system for the removal of multiple contaminants with opposite surface charge.

## Figures and Tables

**Table 1:** The results of the fluoride adsorption study using Al-CNF (the calculated *Q_m_* is 43.3 mg).

Calc. Eqlbrm. Con. of Fluoride, *C_e_* (ppm F^−^)	Con. of Water + Fluoride Sol. 1:1 (ppm F^−^)	Con. of Al-CNF (0.0760 wt%) + Fluoride Solution 1:1 (3 mL each) (ppm F^−^)	Diff. in Con. (ppm)	Percent Removal (%)	Fluoride Removed (mg)	Quantity of Al-CNF (g)	*Q_e_* (mg/g)	*C_e_*/*Q_e_*
0.625	0.4072	0.04239	0.3648	89.6	0.002189	0.00228	0.960	0.651
6.25	6.040	4.954	1.086	18.0	0.006516	0.00228	2.86	2.19
62.5	65.61	59.09	6.52	9.9	0.0391	0.00228	17.2	3.64
500	481.6	467.1	14.5	3.0	0.0870	0.00228	38.2	13.1
2500	2243	2227	16	0.7	0.096	0.00228	42.1	59.3

**Table 2:** The maximum adsorption capacity values (*Q_m_*) of varying adsorbents and Al-CNF.

Adsorbent	Maximum Adsorption Capacity (mg/g)	Reference
Alum sludge	5.4	[21]
Activated alumina (γ-Al_2_O_3_)	16.3	[12]
Alum-impregnated activated alumina	40.7	[22]
Quick lime	16.7	[23]
Schwertmannite	55.3	[24]
Chitosan-based mesoporous alumina	8.3	[25]
Glutaraldehyde-crosslinked calcium alginate	73.6	[26]
Amorphous alumina supported on carbon nanotubes	28.7	[27]
Al-CNF	43.6	This study

**Table 3:** The pH effect on the solvation of aluminum from Al-CNF after fluoride removal.

pH	Dissolved Al^3+^ (ppb)
Acidic	560
Basic	11.8
Neutral	2.7

**Table 4:** Experimental data for Basic Red 2 removal using flocculated fluoride-adsorbed Al-CNF.

Calc. Equilibrium Con. of B.R.2, *C_e_* (ppm)	Absorbance Measured at 518 nm of Water + B.R.2 Solution 1:1 (3 mL Each)	Absorbance Measured at 518 nm of Flocculated Al-CNF (0.1264 wt%) + B.R.2 Sol. 1:1 (3 mL Each)	Con. of Flocculated Al-CNF (0.126 wt%) + B.R.2 Sol. 1:1 (3 mL each)	Conc. Diff. (ppm)	Eff. (%)	B.R.2 Removed (mg)	Quantity of Flocculated Al-CNF (g)	*Q_e_* (mg/g)	Log *C_e_*	Log *Q_e_*
25	0.1220	0.0147	13.51	11.49	46.0	0.035	0.00379	9.090	1.398	0.959
50	0.2447	0.0220	15.06	34.94	69.9	0.105	0.00379	27.64	1.699	1.442
100	0.5039	0.0354	17.91	82.09	82.1	0.246	0.00379	64.94	2.000	1.813
250	1.1850	0.0786	27.11	222.89	89.2	0.669	0.00379	176.3	2.398	2.246
500	2.2398 *	0.1411	40.40	459.60	91.9	1.379	0.00379	363.6	2.699	2.561
1000	4.6762 *	0.2340	60.17	939.83	94.0	2.819	0.00379	743.5	3.000	2.871
2500	10.9905 *	0.2787	69.68	2430.32	97.2	7.291	0.00379	1923	3.398	3.284

* Corrected for Dilution.

## Data Availability

The original contributions presented in the study are included in the article, further inquiries can be directed to the corresponding author.
